# Management of Hypertension in Intrapericardial Paraganglioma

**DOI:** 10.1155/2014/812598

**Published:** 2014-02-13

**Authors:** Nicola Rotolo, Andrea Imperatori, Alessandro Bacuzzi, Valentina Conti, Massimo Castiglioni, Lorenzo Dominioni

**Affiliations:** ^1^Center for Thoracic Surgery, Department of Surgical and Morphological Sciences, University of Insubria, Via Guicciardini 9, 21100 Varese, Italy; ^2^Department of Anaesthesiology, Ospedale di Circolo, Via Guicciardini 9, 21100 Varese, Italy

## Abstract

Functioning paraganglioma is extra-adrenal catecholamine-secreting tumours that may cause secondary hypertension. Primary intrapericardial paragangliomas are very rare and are located adjacent to the great vessels or heart, typically near the left atrium. These tumours are an exceptionally uncommon finding during the investigation of refractory hypertension. However, in recent years, intrapericardial paragangliomas have been diagnosed incidentally with increased frequency, due to the extensive use of radiologic chest imaging. The mainstay of treatment of functioning intrapericardial paraganglioma is surgical removal, which usually achieves blood pressure normalization. Due to the locations of these tumours, the surgical approach is through a median sternotomy or posterolateral thoracotomy, and manipulation-induced catecholamine release may cause paroxysmal hypertension. Typically in these patients, blood pressure fluctuates dramatically intra- and post-operatively, increasing the risk of cardiovascular complications. We review here the current modalities of perioperative fluid and hypotensive drug administration in the setting of surgery for functioning intrapericardial paraganglioma and discuss the recently proposed paradigm shift that omits preoperative preparation.

## 1. Introduction

Hypertension is predominantly essential, but in 15% of cases it is secondary to renal or endocrine diseases [1]. Pheochromocytoma and extra-adrenal paraganglioma are rare chromaffin tumours arising from neural crest tissue that develops into sympathetic and parasympathetic paraganglia throughout the body. These tumours may secrete large amounts of catecholamines and represent an uncommon identifiable cause of resistant hypertension [2]. The World Health Organization classification uses the term *pheochromocytoma* only for tumours of adrenal medulla origin and the term *paraganglioma* for similar lesions that arise from other locations [3].

Catecholamine-secreting tumours occur in less than 0.1% of the hypertensive population; the peak incidence is between the third and fifth life decades, with both genders being equally affected. Twenty-five percent of pheochromocytoma and paraganglioma occur in the setting of familial syndromes (SDH gene mutations; MEN 2A/B; von Hipple-Lindau; Neurofibromatosis I; Carney triad). Thus, family history should be carefully investigated in patients with catecholamine-secreting tumours, and all relatives should be screened for hereditary syndromes [4–6]. Most paragangliomas are solitary, but they tend to be multicentric in hereditary syndromes; in an institutional series of 12 cases, 25% were multicentric [5].

## 2. Incidence and Prognosis of Intrapericardial Paraganglioma

Less than 2% of reported paraganglioma are located in the chest and intrapericardial paraganglioma (IP) is even rarer [5].

Most IPs are large, ranging in size from 3 to 8 cm [6, 7]; in the 72 cases listed in Table 1, the average tumour diameter was about 5 cm. This suggests that the tumour mass grows undetected for long time before becoming symptomatic.

Little is known regarding long-term survival of IP patients, as long-term follow-up studies are not available.

The rarity of IP is well documented, only 30 cases being reported until 1992 [8]. We reviewed the medical literature subsequently published and found that over the two-decade period 1994–2013 three institutional series [5, 6, 9] including altogether 33 cases and 39 other individual case reports of IP were described (Table 1). In total, 102 cases of IP have been reported in the literature to date.

## 3. Clinical and Diagnostic Features

Arterial hypertension is the most common clinical presentation of IP; about 10% of reported paragangliomas are clinically silent (Table 1), the tumour being diagnosed incidentally after chest radiography, computed tomography (CT), or magnetic resonance (MR) imaging performed for unrelated reasons. More frequently the disease presents with paroxysmal symptoms of excess catecholamine production, which include hypertension, headache, palpitations, tremor, and facial pallor. Hypertension episodes are variable in frequency, severity, and duration and are difficult to treat. The hypertension crisis may induce arrhythmia and myocardial ischemia and may even be fatal. Bursts of catecholamine secretion can be provoked by a variety of events, which include accidental or surgical trauma, anaesthesia induction, invasive procedures, and eating food with high level of tyramine (e.g., red wine, chocolate, and cheese).

About 20% of IPs are nonfunctioning (Table 1) and may present as a mass with symptoms related to compression of other organs, or they are incidentally discovered during imaging studies [9, 10].

The diagnosis of functioning paraganglioma requires biochemical testing to document elevated catecholamine secretion and is generally obtained by determining the plasma level of fractionated metanephrines during hypertensive crisis and the 24-hour urine normetanephrine level.

Imaging exams to localize the tumour and its metastases include CT and MR imaging, coupled with 123-I-metaiodobenzylguanidine (MIBG) scintigraphy. These exams provide anatomical and functional information with good sensitivity and specificity [11].

Some IP cases have been incidentally identified by chest radiography, as an enlargement of mediastinum or splaying of the carina by the tumour mass [5]. On CT scan IP typically appears as a well-enhancing mass, predominantly located in the posterior mediastinum or in the aortic-pulmonary window. The most frequent location is in close proximity to the left atrium (Table 1). CT and MR images allow to clarify tumour mass relationships with surrounding mediastinal structures and are of great importance for planning surgical resection.

## 4. Treatment

Surgical resection is the mainstay of treatment for benign and malignant IP. Resection must be completed with minimal tumour manipulation to prevent hypertensive crisis and tumour seeding. Control of tumour vascular supply requires adequate operative field exposure to avoid injury to surrounding organs. The operative access to the mediastinum depends on location and size of the lesion, as well as on the adjacent structures involved. Generally, the surgical approach to IP is with sternotomy, as cardiopulmonary bypass (CPBP) is usually planned for tumour removal. Less commonly, IP can be resected without CPBP, through left or right thoracotomy, depending on tumour location. If size and location of the lesion are favourable, the approach can also be with minimally invasive videothoracoscopic technique.

Patients with unresectable or metastatic disease may be treated with chemotherapy, radiofrequency thermoablation, cryoablation, and catecholamine blockade [11].

Perioperative management of IP patients is a challenge for anaesthesiologists and surgeons. Over the last 50 years, shared management of the patient involving also endocrinologists and cardiologists translated into a marked reduction of perioperative mortality from 40–60% to 0–6% [12].

## 5. Perioperative Management of Hypertension

In preparation for surgery, a detailed medical history is essential, along with physical examination, complete laboratory exams, and evaluation by cardiologist and anaesthesiologist. It is important to detect the presence of a cardiomyopathy or coronary artery disease by ECG, echocardiography, and coronary angiography, if necessary. However, for young patients without history of heart disease, it is debated whether preoperative ECG only is sufficient [13]. Echocardiography should be done to assess the presence of hypertrophic, dilated or Tako-Tsubo cardiomyopathy. This imaging technique is also useful to localize cardiac paraganglioma [14].

If hypertrophic cardiomyopathy as a result of chronic norepinephrine-induced hypertension is found, it is mostly symmetric and concentric [12, 13]. Dilated cardiomyopathy has also been described.

An interesting finding in patients with functioning chromaffin tumours is the stress-related Tako-Tsubo cardiomyopathy, also called left ventricular apical ballooning syndrome. The pathophysiology of stress-induced and chromaffin tumour-induced cardiomyopathy is believed to be similar and mediated by catecholamines causing myocardial stunning [15]. Typically in Tako-Tsubo disease, the decreased ejection fraction caused by myocardial alterations undergoes spontaneous recovery. Common electrocardiographic findings in patients with catecholamine-secreting tumours are high QRS amplitudes with abnormal R, changes in ST-segment and T waves, and prolongation of the Q-Tc interval [13].

Preoperative medical management to block the deleterious effects of excess catecholamine release and to allow plasma volume expansion is recommended [16]. Although *α*-adrenoceptor antagonists, calcium-channel blockers, or angiotensin-receptor blockers have all been recommended, there are no evidence-based guidelines on the preferred drugs for preoperative catecholamine blockade. Consequently, there are widely ranging practices and international differences in perioperative pharmacologic management and approved therapies. Importantly, *β*-blockers should be used only after adequate pretreatment with *α*-antagonists. Moreover, volemia expansion is also widely recommended before and after surgery [16].

### 5.1. Preoperative Optimization of Blood Pressure

Special preparation of the patient undergoing surgical removal of paraganglioma is necessary once clinical, instrumental, and laboratory evaluations are completed.

Without preoperative medical treatment, induction of anaesthesia or other stimuli can cause a hypertensive crisis, cardiac arrhythmias, and infarction or stroke, due to massive catecholamine release. Pharmacological treatment should be instituted for 1 to 2 weeks before surgery, to optimize cardiovascular function by relaxation of the constricted vasculature, expansion of the reduced plasma volume, and normalization of blood pressure. Normalization of blood volume reduces the risk of prolonged hypotension after tumour removal [16].

Preoperative antihypertensive therapy is useful in patients with sustained or paroxysmal hypertension. In this setting, interventions are focused on maintaining adequate plasma volume and on lowering blood pressure using *α*- and *β*-blockers. Alpha-adrenergic blockade should be started prior to *β*-adrenergic blockade to prevent acute hypertensive crisis. Alpha-blockade is generally initiated 7 to 10 days before surgery, using different drugs (phenoxybenzamine, prazosin, or doxazosin) to achieve a systolic blood pressure below 120 mmHg when seated and below 90 mmHg when standing. It is recommended that no blood pressure >160/90 mmHg values should be evident 24 hours before surgery, and an orthostatic hypotensive response (blood pressure >80/45 mmHg) should be present. Moreover, no electrocardiographic ST/T segment changes at least for 1 week should be evident [17]. After reaching these blood pressure values, it is possible to initiate *β*-blocker administration, using low dosages to reduce the risk of negative inotropy [18]. Magnesium sulphate has also been shown useful to control blood pressure, when haemodynamic stability is difficult to achieve [18].

### 5.2. Intraoperative Management

During the operative period, it is essential to closely monitor cardiovascular function by electrocardiogram and to assess urine output, pulse oxinmetry, capnography, and body temperature. It may be necessary to monitor cerebral function with electroencephalography, if the patient has a recent history of cerebral infarction [19].

Cardiovascular monitoring requires an intra-arterial catheter and a central venous catheter to respond quickly to haemodynamic changes with vasoactive agents or fluid administration [13]. Pulmonary capillary wedge pressuremonitoring may be useful because of discrepancy between right-sided and left-sided filling pressures, but the routine use of pulmonary catheters remains controversial. Transesophageal echocardiography is useful to optimise intravenous fluid administration and to assess perioperative cardiac function [13, 20].

### 5.3. Anaesthetic Technique

General anaesthesia is the most commonly chosen technique. Intravenous or inhalation agents have been used with success, but drugs that stimulate the sympathetic system responses or that may cause mechanical stimulation of the tumour by fasciculations, such as succinylcholine, should be avoided. Deep anaesthesia to inhibit cardiovascular response is more important than the specific agent used [13].

Several drugs are available to control blood pressure during tumour resection. Sodium nitroprusside should be used in patients with a history of acute myocardial infarction or congestive heart failure, although this drug has potential for overshoot hypotension [21]. Phentolamine is *α*-adrenergic antagonist that can be given intravenously as continuous infusion or as boluses of 1 to 2 mg; it can cause tachycardia if the patient is not receiving *β*-blockers. Calcium channel blockers have some advantages over sodium nitroprusside, such as less risk of deep hypotension, no rebound hypertension, more controlled heart rate, and absence of cyanide toxicity [12]. Fenoldopam, a dopamine-1 receptor agonist that causes peripheral vasodilation and increases renal blood flow, may also be used.

After tumour removal, it is essential to control hypotension that may result from inadequate intravascular volume, residual *α*-adrenergic antagonist effect, alteration of venous capacitance and haemorrhage. Priority should be given to volume replacement, which seems to be the main factor responsible for reduction of intraoperative mortality in chromaffin tumour surgery [12, 22]. In addition to blood pressure control, it is necessary to carefully monitor blood glucose level, because hypoglycemia may develop after tumour removal, due to rebound hyperinsulinism, as the inhibitory effect of norepinephrine on insulin secretion is eliminated.

**Figure 1:**
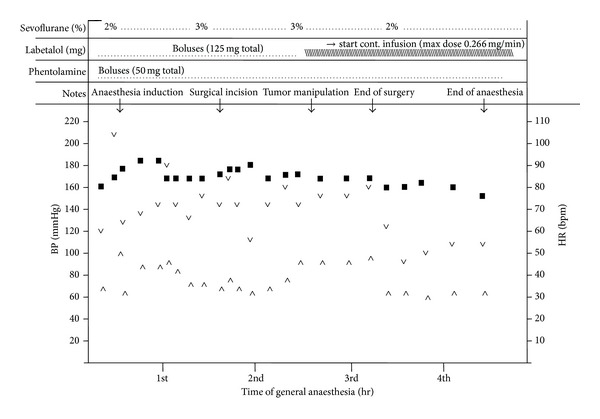
Anaesthesia management during surgery. (■) heart rate; (∨) systolic blood pressure; (∧) diastolic blood pressure. As noted, during critical steps of surgery, it has been necessary to administer a greater amount of sevoflurane, in association with multiple boluses of beta-blocker. Total labetalol administered was 125 mg + continuous infusion 2 mg/mL with maximum administration of 0.266 mg/min. Phentolamine was administered by boluses of 1-2 mg (50 mg total).

**Figure 2:**
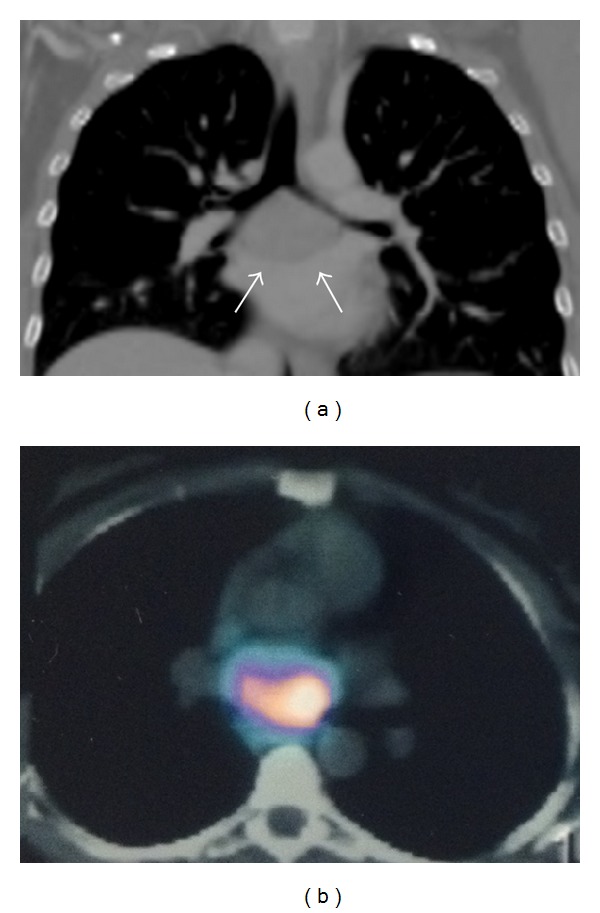
(a) Coronal view of chest computed tomography, revealing an intrapericardial 55 mm mass located at the roof of the left atrium (arrows). (b) ^123^I-metaiodobenzylguanidine chest scan showing high uptake by the intrapericardial mass.

**Figure 3:**
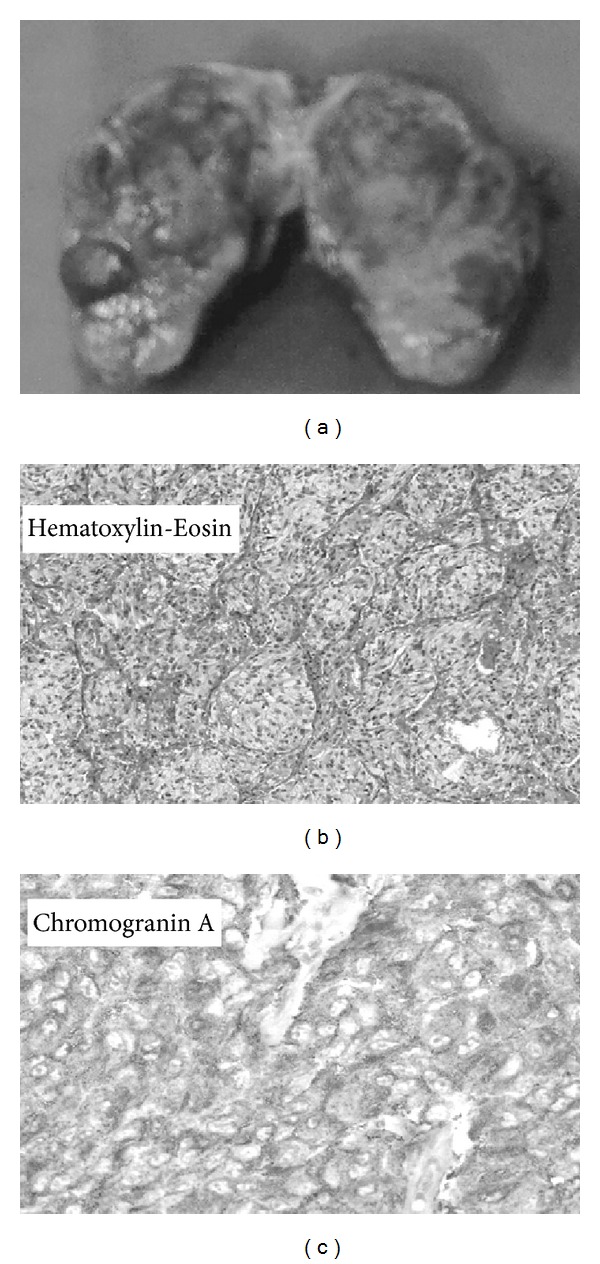
(a) Resected specimen (55 × 45 mm pseudocapsulated mass). Histology showed (b) paraganglioma with a nesting pattern (hematoxylin-eosin, ×200) and (c) strong cytoplasmic immunoreactivity for chromogranin A (×200).

To illustrate the intraoperative management of anaesthesia, we report in Figure 1 the details of *β*-blocker and *α*-adrenergic antagonist administration in a case undergoing surgery for removal of IP at our institution. The patient, a 34-year-old woman with a history of recent episodes of headache, palpitation, and flushing, had hypertension (180/90 mm Hg) poorly controlled by ramipril. She was diagnosed with a functioning IP localized below the left atrial roof (Figure 2). Tumour resection was carried out through right thoracotomy without CPBP. A hypertensive crisis developed at the time of general anaesthesia induction (210/100 mm Hg). During the critical steps of surgery, it was necessary to administer a greater amount of the anaesthetic drug sevoflurane, in association with multiple boluses of *α*- and *β*-blockers. Total labetalol boluses administered amounted to 125 mg, in addition to continuous infusion of labetalol 2 mg/mL (maximum administration of 0.266 mg/min). Phentolamine was administered in boluses of 1-2 mg (50 mg total) (Figure 1). After successful resection of the intrapericardial mass, which was histologically shown to be a chromogranin A-positive paraganglioma (Figure 3), the patient was discharged normotensive [23].

Some authors have questioned the validity of systematic preoperative optimization of blood pressure and volume expansion, drawing attention to the lack of evidence-based studies [22]. However, there is abundant literature to support a proper pharmacological preparation in all patients with functioning paraganglioma and pheochromocytoma, especially in subjects with cardiovascular risk.

We experienced that preoperative medical preparation of cardiac paraganglioma patients is critical. Alpha-adrenergic blockade (phenoxybenzamine or phentolamine) is usually started at the time of diagnosis and it is carried on preoperatively under close blood pressure monitoring, to prevent cardiovascular complications that may occur during surgery due to excess catecholamine secretion. These complications include hypertensive crisis, arrhythmia, myocardial infarction, and pulmonary edema.

The goal of intraoperative pharmacologic therapy is to prevent sudden rise and fall of blood pressure. Beta-adrenergic and calcium channel blockade can be used as adjuncts when blood pressure or tachycardia cannot be controlled [6].

At the time of tumour vascular supply control, rapid decrease of catecholamine level and rebound hypotension should be expected [24], and preoperative volume expansion helps in preventing it [6].

## 6. Surgical Outcome and Follow-Up

After surgical removal of IP, approximately 80% of patients become normotensive (Table 1). Persistent hypertension may be due to incomplete tumour resection or metastatic disease. The other 20% remain hypertensive without biochemical evidence of residual tumour, due to associated essential hypertension or to acquired renovascular changes [24].

Tumour recurrence in case of incomplete resection may occur; moreover, approximately 10% IPs were found to be malignant; therefore, systematic follow-up is recommended. There is no general agreement on the method and frequency of follow-up after paraganglioma resection. Long-term follow-up is recommended with blood pressure measurements and periodic determination of urinary metanephrines; if necessary, 123-I-MIBG scintigraphy and CT imaging should be obtained [16].

## 7. Conclusion

IP is a rare tumour that may cause secondary hypertension. Surgical removal is the gold-standard treatment and normalizes blood pressure in about 80% of hypertensive cases; 20% remain hypertensive, likely due to associated essential hypertension. Preoperative medical preparation is critical to prevent cardiovascular complications that may result from excess catecholamine secretion during anaesthesia induction and surgical manipulation of the tumour. Alpha-adrenergic blockade and *β*-blockers are usually started at the time of diagnosis and this treatment is carried on perioperatively under close monitoring of blood pressure. Due to the rarity of functioning paraganglioma, systematic perioperative optimization of blood pressure and volume expansion has not been validated by randomized studies. However, there is abundant literature to support the value of pharmacological preparation and volume expansion to prevent cardiovascular complications.

## Figures and Tables

**Table 1:** Published case reports of intrapericardial paraganglioma in 1994–2013.

Authors	Year	No. of Pts	Symptoms	Tumour location	Catechol. secretion increase	Treatment	Hypertension after surgery	CPBP
Gonzale-Lopez et al. [25]	2013	1	Hered. Synd.	Retroc.	Yes	Surg.	No	Yes
Liu et al. [26]	2013	1	HT.	Interatrial.	Yes	Surg.	No	Yes
Wang et al. [27]	2013	1	HT.	R.A.	n.s.	Surg.	No	Yes
Tracy and Wein [28]	2013	1	Hered. Synd.	R.A.	Yes	n.s.	n.s.	n.s.
Ramlawi et al. [9]	2012	7	5/7 Pts	71% L.A.	No	Surg.	No	Yes
Marshall et al. [29]	2012	1	HT.	R.A.	Yes	Surg.	No	Yes
Huo et al. [30]	2012	2	Palpitation	L.A.; R.A.	Yes	Surg.	No	Yes
Aki et al. [31]	2012	1	Chest pain	INT. SEPT.	n.s.	Surg.	n.s.	Yes
Beroukhim et al. [32]	2012	1	Palpitation	Pulm. Art.	n.s.	Surg.	n.s.	n.s.
Imperatori et al. [23]	2011	1	HT.	INT. PERIC.	Yes	Surg.	No	No
Al-Githmi et al. [33]	2010	1	Chest pain	Aortic root	n.s.	Surg.	n.s.	Yes
Cong et al. [34]	2011	1	HT.	INT. PERIC.	Yes	Surg.	No	n.s.
Ceresa et al. [35]	2010	1	HT.	L.A.	Yes	Surg.	No	Yes
Petersen et al. [36]	2010	1	Palpitations	L.A.	Yes	Surg.	n.s.	Yes
Gómez et al. [37]	2010	1	Resp. infect.	L.A.	n.s.	Surg.	No	No
Rana et al. [38]	2009	1	HT.	INT. PERIC.	Yes	Surg.	n.s.	No
Lorusso et al. [7]	2009	1	Chest pain	L.V.	No	Surg.	n.s.	Yes
Zhou et al. [39]	2009	1	HT.	R.A.; R.V.	Yes	Surg.	No	Yes
Tahir et al. [40]	2009	1	Chest pain	R.A.	No	Surg.	n.s.	Yes
Thomas et al. [41]	2009	1	HT.	R.A.	Yes	Surg.	No	n.s.
Alghamdi et al. [42]	2009	1	None	INT. PERIC.	n.s.	Surg.	No	n.s.
Lee et al. [43]	2009	1	None	INT. PERIC.	n.s.	Surg.	n.s.	n.s.
Brown et al. [6]	2008	14	14/14 HT.	Heart	Yes	Surg.	7/14	2/14
Vicente et al. [10]	2008	1	Wheezing	L.A.	No	Surg.	n.s.	No
Hawari et al. [44]	2008	1	Chest pain	R.V.	No	Surg.	No	Yes
Ali et al. [45]	2007	1	HT.	INT. PERIC.	n.s.	Surg.	n.s.	n.s.
Maxey et al. [46]	2007	1	Palpitations	INT. SEPT.	n.s.	Surg.	n.s.	Yes
Yuan et al. [47]	2007	1	HT.	R.A.	Yes	Surg.	No	n.s.
Imren et al. [48]	2007	1	HT.	L.A.	n.s.	Surg.	n.s.	Yes
Jimenez et al. [49]	2005	1	Palpitation	L.A.	n.s.	CHT	No	No
Turley et al. [50]	2005	1	Chest pain	INT. SEPT.	No	Surg.	No	Yes
Moorjani et al. [51]	2004	1	HT.	L.A.	Yes	Surg.	No	Yes
Lupinski et al. [52]	2004	1	HT.	R.V.	Yes	Surg.	Yes	Yes
Boumzebra et al. [53]	2002	1	None	INT. PERIC.	n.s.	Surg.	n.s.	Yes
Tekin et al. [54]	2000	1	Dysphagia	L.A.	No	Surg.	n.s.	n.s.
Pickering et al. [55]	2000	1	HT.	R.V.	Yes	Surg.	No	Yes
Dresler et al. [56]	1998	1	HT.	L.A.	n.s.	Surg.	n.s.	Yes
Hamilton et al. [5]	1997	12	HT.	83% L.A.	Yes	11/12 Surg.	n.s.	2/11
Cane et al. [57]	2012	1	n.s.	INT. SEPT.	n.s.	Surg.	n.s.	n.s.
Casanova et al. [58]	1996	1	HT.	INT. PERIC.	Yes	Surg.	No	Yes
Williams et al. [59]	1994	1	n.s.	INT. PERIC.	n.s.	Surg.	No	No
Gomi et al. [60]	1994	1	HT.	L.A.	Yes	Surg.	No	Yes

Pts: patients; CPBP: cardiopulmonary bypass; Hered. Synd.: hereditary syndrome; Retroc.: retrocardiac; HT.: hypertension; n.s.: not specified; R.A.: right atrium; L.A.: left atrium; INT. SEPT.: interatrial septum; INT. PERIC.: intrapericardial; L.V.: left ventricular; R.V.: right ventricular; CHT: chemotherapy.
